# Comprehensive transcriptome resource for response to phytohormone-induced signaling in *Capsicum annuum* L.

**DOI:** 10.1186/s13104-020-05281-1

**Published:** 2020-09-18

**Authors:** Junesung Lee, Jae-Young Nam, Hakgi Jang, Nayoung Kim, Yong-Min Kim, Won-Hee Kang, Seon-In Yeom

**Affiliations:** 1 Department of Agricultural Plant Science, Division of Applied Life Science (BK21), Gyeongsang National University, Jinju, 52828 Republic of Korea; 2 Institute of Agriculture & Life Science, Gyeongsang National University, Jinju, 52828 Republic of Korea; 3 Genome Engineering Research Center, Korean Bioinformation Center, Korea Research Institute of Bioscience and Biotechnology, Daejeon, 34141 Republic of Korea

**Keywords:** Transcriptome, Phytohormone signaling, Environmental stresses, *Capsicum annuum*

## Abstract

**Objectives:**

Phytohormones are small signaling molecules with crucial roles in plant growth, development, and environmental adaptation to biotic and abiotic stress responses. Despite several previously published molecular studies focused on plant hormones, our understanding of the transcriptome induced by phytohormones remains unclear, especially in major crops. Here, we aimed to provide transcriptome dataset using RNA sequencing for phytohormone-induced signaling in plant.

**Data description:**

We used high-throughput RNA sequencing profiling to investigate the pepper plant response to treatment with four major phytohormones (salicylic acid, jasmonic acid, ethylene, and abscisic acid). This dataset yielded 78 samples containing three biological replicates per six different time points for each treatment and the control, constituting 187.8 Gb of transcriptome data (2.4 Gb of each sample). This comprehensive parallel transcriptome data provides valuable information for understanding the relationships and molecular networks that regulate the expression of phytohormone-related genes involved in plant developments and environmental stress adaptation.

## Objective

Plants are sessile beings, which are exposed to various attacks from the environment involving biotic/abiotic stress conditions [1, 2]. Besides, plants interact with positive effects from plant-associated microbes which induce phytohormones so that strengthen plants to withstand stresses. In response to these physiological processes, different signaling pathways of plant hormones are activated. Infection of plants with diverse pathogens results in changes in the level of various phytohormones. Three phytohormones—salicylic acid (SA), jasmonic acid (JA) and ethylene (ET), are known to regulating plant defense responses against various pathogens, pests and abiotic stresses. Abscisic acid (ABA) exert opposite defense effect from these hormones, but can also enhance disease resistance [3, 4]. These phytohormones tend to act interdependently through complex antagonistic or synergistic interactions [5]. These relationships reveal that important networks of phytohormone crosstalk exist to mediate physiological processes such as biotic, abiotic stress tolerance, and plant growth.

Despite several previously reported molecular studies focused on plant hormones, the transcriptome information of phytohormones remains unclear, especially in major crops [6, 7]. Recently a few genes and gene families regulated by phytohormones have been identified in pepper [8–10], but a time-series investigation of the well-regulated transcriptome network has yet to be performed. Accordingly, this study aimed to provide transcriptome dataset using RNA sequencing (RNA-seq) for transcriptome dataset of phytohormone-induced signaling in pepper plant. In this study, we performed transcriptome analysis of pepper treated with four major phytohormones, namely SA, JA, ET, and ABA, at six time points. Total 78 RNA samples were subjected to RNA-seq by constructing strand-specific RNA libraries, and 187.8 Gb of transcriptome data were produced. These transcriptomic profiles will contribute to our understanding of the phytohormone-induced signaling pathways involved in response to environmental stresses and plant development in pepper and other crops.

## Data description

### Plant materials and treatment

Pepper seeds (*C. annuum* cv. Bukang) were sown on petri dish lined with a wet tissue layer for 2 weeks. After germination, seedlings were transplanted into a 32-cell plug seedling tray and grown at 24 ± 1 °C with an alternating 16-h light/8-h dark photoperiod. At the 6-true-leaf stage, pepper plants were sprayed with 5 mM sodium salicylate (SA), 100 μM methyl jasmonate (JA), 5 mM ethephone (ET), 100 μM ( ±)-ABA, or distilled water (mock) [11–14]. Each was treated and incubated in the growth chamber separately to avoid cross-contamination. After treatment, the third or fourth leaf was collected at 0, 1, 3, 6, 12, and 24 h post-inoculation, and frozen with liquid nitrogen immediately prior to storage at − 80 °C. Each treatment time point was performed for three biological replicates, and leaves from four healthy plants were gathered for a replicate.

### RNA extraction, library construction, and sequencing

Following phytohormone inoculation, total RNA from pepper leaves was extracted using Trizol reagent (Ambion, USA) according to the manufacturer’s instructions. To confirm the phytohormone response for each treatment, semi-quantitative RT-PCR was performed using gene primers such as SA (*CaPR1*), JA (*CaPin2*), ET (*CaACO*), and ABA (*CaWRKY40*) [13–16]. Expression levels were normalized with the *CaActin* [17] and the mock group was used as a control (Data file 1).

Samples of total RNA (5 μg) were used to prepare strand-specific libraries as described previously [18, 19]. In brief, from each total RNA, the Poly-(A) RNA was captured and fragmented by the size of 300 to 400 bp. The RNA fragments were generated second-strand cDNA, and then performed end-repair, dA tailing, adapter ligation and PCR amplification. We generated total 78 cDNA libraries consisting of four treatment groups and a mock control group, for transcriptome profiling. Strand-specific RNA libraries were sequenced using the 151nt paired-end on the HiSeq2500 platform (Illumina, USA) at Macrogen Corporation (Korea) (Table 1).

**Table 1:** Overview of data files/data sets

Label	Name of data file/data set	File types (file extension)	Data repository and identifier (DOI or accession number)
Data set 1	Comprehensive transcriptome profiling for response to phytohormone-induced signaling in *Capsicum annuum* L.	fastq (.fastq)	Sequence Read Archive (https://identifiers.org/ncbi/insdc.sra:SRP265260)
Data set 2	Comprehensive transcriptome profiling for response to phytohormone-induced signaling in *Capsicum annuum* L.	text (.txt)	Gene Expression Omnibus (https://identifiers.org/geo:GSE149037)
Data file 1	Data file 1. Schematic workflow of experimental design and bioinformatics analysis in this study	Adobe acrobat file (.pdf)	Figshare (https://doi.org/10.6084/m9.figshare.12319337.v6)
Data file 2	Data file 2. Statistical summary of RNA-seq with SRA accession numbers for each treatment	MS Excel file (.xlsx)	Figshare (https://doi.org/10.6084/m9.figshare.12319337.v6)
Data file 3	Data file 3. Quality assessment metrics for RNA-seq data	Adobe acrobat file (.pdf)	Figshare (https://doi.org/10.6084/m9.figshare.12319337.v6)
Data file 4	Data file 4. Normalized FPKM	MS Excel file (.xlsx)	Figshare (https://doi.org/10.6084/m9.figshare.12319337.v6)

### Quality control and quantification of gene expression

The adapter filtering and quality trimming was performed on a total of 78 RNA libraries using the Cutadapt and Trimmomatic programs, respectively [20–22]. The read length of each sample was filtered by QC and the read length was 28.87–6.07 Gb (Data file 2). After filtering, the quality of pre-processed reads were checked using FastQC [23] and the output was merged using MultiQC (Data file 3) [24]. Read mapping was carried out with the *C. annuum* ‘CM334’ reference genome v.1.6 (https://peppergenome.snu.ac.kr) using Hisat2 [25]. Transcriptome quantification was performed using HTseq-count [26] to calculate the read counts. The clean reads were mapped to the coding DNA sequence with 65.75–70.36% and the genome with 92.13 –96.04% (Data file 2). Raw read count was normalized to FPKM and visualized with the distribution (Data files 3, 4). The principal component analysis (PCA) with normalized data was used to examine sample variation (Data file 3) [27, 28]. The comparisons between PC1 and PC2 (SA, ET) or PC1 and PC3 (JA, ABA) indicated that the mock and phytohormone-treated groups were separated clearly.

## Limitations

Raw data was deposited in NCBI, and quality filtering is required before use. The transcriptome data was generated using *C. annuum* cv. Bukang, and read mapping was carried out with *C. annuum* cv. CM334 reference genome.

## Data Availability

The RNA-seq library of 78 samples are publicly available from the Sequence Read Archive at accession number https://identifiers.org/ncbi/insdc.sra:SRP265260 [29]. The quantified transcriptome expression data was deposited in the NCBI Gene Expression Omnibus database with an accession number of https://identifiers.org/geo:GSE149037 [30]. The combined additional files and information generated in this study have been uploaded to figshare, with accession number https://doi.org/10.6084/m9.figshare.12319337.v6 [31].

## Abbreviations

ABA: Abscisic acid
ET: Ethylene
FPKM: Fragments per kilobase of transcripts per million mapped reads
PCA: Principal component analysis
QC: Quality control
RNA-seq: RNA sequencing
RT-PCR: Reverse transcription polymerase chain reaction
SA: Salicylic acid
